# Retinal Cell Biology in Health and Disease

**DOI:** 10.3390/cells13040297

**Published:** 2024-02-06

**Authors:** Hossein Ameri

**Affiliations:** Department of Ophthalmology, USC Roski Eye Institute, Keck School of Medicine, University of Southern California, Los Angeles, CA 90033, USA; ameri@med.usc.edu

The intricate network of cells and processes that govern retinal health has long been a subject of fascination and intensive study within the scientific community. In recent breakthroughs, a series of papers have shed light on various aspects of ocular physiology, presenting findings that deepen our understanding of eye health and open new avenues for therapeutic interventions. From mitophagy in astrocytes to membrane attack complex-mediated cell death, these studies explore diverse facets of retinal cell biology, contributing to the broader landscape of vision research.

One of these compelling revelations comes from a study on mitophagy in astrocytes by Yazdankhah et al., which emphasizes the crucial role of mitophagy in preserving mitochondrial function and maintaining the health of the optic nerve [1]. The optic nerve, responsible for transmitting visual information from the retina to the brain, relies on the intricate interplay between cells, and the role of mitophagy in astrocytes highlights the importance of cellular maintenance mechanisms in preserving optic nerve function. This research not only enhances our comprehension of optic nerve health but also suggests potential therapeutic strategies for conditions affecting mitochondrial function such as Leber’s hereditary optic neuropathy.

Another noteworthy discovery pertains to the interaction between Disheveled-1 and Claudin-5 in the context of Norrin-induced endothelial barrier restoration, which was studied by Diaz-Coranguez et al. [2]. Understanding the molecular mechanisms underlying endothelial barrier function is pivotal, especially in the context of vascular endothelial growth factor overexpression in conditions such as diabetic retinopathy and retinal vein occlusion. This research not only identifies a key player in the restoration process but also unveils potential targets for therapeutic intervention in conditions involving endothelial barrier dysfunction.

The synaptic ribbon of rod photoreceptor synapses takes center stage in the study of Rabconnectin-3α/DMXL2 enrichment by Dittrich et al. [3]. The local concentration of this protein at the synaptic ribbon underscores its significance in the intricate machinery governing neurotransmission in the retina. Insights into the molecular intricacies of photoreceptor synapses provide a foundation for understanding visual signal processing and may pave the way for novel therapeutic approaches in vision-related disorders.

Shifting the focus to cellular signaling pathways, the research on Peptide Lv by Pham et al. stands out due to its exploration of the trafficking and membrane insertion of K_Ca_3.1 [4]. Unraveling the complex interplay between Protein Lv and MEK1–ERK and PI3K–Akt signaling pathways in this context not only deepens our understanding of cellular regulation but also presents potential targets for modulating ion channel activity in retinal vascular endothelial cells, opening new avenues for therapeutic intervention in conditions involving pathogenic angiogenesis such as wet age-related macular degeneration.

The anatomical success of reattaching a detached retina does not guarantee a full restoration of vision to its pre-detachment level. The visual recovery is inversely related to the duration of the detachment. The study conducted by Townes-Anderson et al. sheds light on the differential impact of retinal detachment on cone and rod synapses [5]. Furthermore, the protective role of Rho kinase inhibition highlights a potential avenue for the preservation of cone synapses and, consequently, visual function in chronic retinal detachment.

Mitochondrial dysfunction takes center stage in Huang et al.’s study, who examined impaired antioxidant responses in retinal pigment epithelial cells associated with RCBTB1-related retinopathy [6]. This research not only provides insights into the pathophysiology of a specific retinal disorder but also underscores the broader significance of mitochondrial health in maintaining retinal function.

Lastly, the involvement of the membrane attack complex in retinal pigment epithelium cell death in Stargardt Macular Degeneration, studied by Ng et. al., unveils a critical aspect of the immune response in ocular degenerative diseases [7]. Understanding the mechanisms underlying cell death in these conditions is crucial for developing targeted therapies to halt or slow down disease progression.

In conclusion, the papers discussed here collectively contribute to a deeper understanding of ocular health, spanning various aspects of cellular and molecular biology. As we unravel the intricacies of mitophagy, synaptic ribbons, signaling pathways, and immune responses in the eye, we pave the way for innovative therapeutic strategies that may revolutionize the treatment of retinal disorders. These findings not only advance our scientific knowledge but also hold the promise of improving the lives of individuals affected by vision-related conditions. The journey towards comprehensive ocular health continues, fueled by the relentless pursuit of knowledge and the hope of transformative medical advancements.
